# Validation of the Acute Physiology and Chronic Health Evaluation (APACHE) II Score in COVID-19 Patients Admitted to the Intensive Care Unit in Times of Resource Scarcity

**DOI:** 10.7759/cureus.34721

**Published:** 2023-02-07

**Authors:** Salomão Fernandes, Rita Sérvio, Patrícia Patrício, Carlos Pereira

**Affiliations:** 1 Intensive Care Unit, Hospital Beatriz Ângelo, Loures, PRT

**Keywords:** apache ii score, severity scores, medical icu, sars-cov-2, covid-19

## Abstract

Introduction

During the coronavirus disease 2019 (COVID-19) pandemic, a high number of patients needed to be admitted to the intensive care units (ICUs). Such a high demand led to periods where resources were insufficient and the triage of patients was needed. This study aims to evaluate the performance of the Acute Physiology and Chronic Health Evaluation (APACHE) II as a predictor of mortality in periods where triage protocols were implemented.

Methods

A single-center, longitudinal, retrospective cohort study was performed on patients admitted to the ICU between January 2020 and December 2021. Patients were divided into two periods: Period 1 (where patients needing ICU admission outnumbered the available resources) and Period 2 (where resources were adequate). The discriminative power of the APACHE II was checked using the receiver operating characteristic (ROC) curves. Calibration was accessed, and survival analysis was performed.

Results

Data from 428 patients were analyzed (229 in Period 1 and 199 in Period 2). The area under the ROC curve (AUROC) was 0.763 for Period 1 and 0.761 for Period 2, reflecting a good discriminative power. Logistic regression showed the APACHE II to be a significant predictor of mortality. The Hosmer-Lemeshow test demonstrated good calibration. The Youden index was determined, and a log-rank test showed a significantly lower survival for patients with higher APACHE II scores in both periods.

Conclusions

The APACHE II score is an effective tool in predicting mortality in patients with COVID-19 admitted to the ICU in a period where resource allocation and triage of patients are needed, paving a way for the future development of better and improved triage systems.

## Introduction

Coronavirus disease 2019 (COVID-19) was declared a pandemic by the World Health Organization on March 11, 2020 [1]. In the following months, high numbers of infected patients accounted for added pressure for healthcare systems and professionals worldwide, including in the intensive care units (ICUs) [2].

Risk scores (also called prognostic scoring systems or severity scores) such as the Acute Physiology and Chronic Health Evaluation (APACHE), Sequential Organ Failure Assessment (SOFA), quickSOFA (qSOFA), and Simplified Acute Physiology Score (SAPS) are widely used as ways to predict outcomes of patients admitted to the ICUs [3].

The APACHE II score was developed as a tool to predict hospital mortality using clinical variables from the first hours post-admission to an ICU as well as information from patients’ characteristics and medical history [4]. Today, the APACHE II is one of the most widely used risk scores [5-7].

So far, several studies have validated APACHE II scores in patients with COVID-19 [8-13]. One review paper looked at studies using traditional risk scores, COVID-19-specific risk scores, and artificial intelligence-generated risk scores during the pandemic [14]. One key finding was that current risk systems generally underestimate mortality in COVID-19 patients. It was also found that the APACHE II has the highest area under the receiver operating characteristic (ROC) curve (AUROC) values of all the traditional risk scores included, being the best one at predicting mortality for COVID-19 patients.

Looking back at the daily reported numbers of COVID-19 patients in Portugal [15] and reports regarding the overall COVID-19 response by the healthcare system and the availability of resources [16], we can observe that during the month of November and December 2020 and January and February 2021, the number of patients in need of intensive care support outnumbered by a large margin the critical values defined by the Portuguese health authorities. That created not only the need to open new temporary intensive care units but also the need for a triage system for better resource allocation.

In the first months of the pandemic and predicting the necessity for patients’ triage, several publications discussed principles regarding resource allocation for patients needing ICU admission [17-20]. Recommendations were made not only based on ethical considerations but also on clinical ones, where clinical decision support systems and risk scores (such as the APACHE II) could be included [21].

So far, there are no studies validating the APACHE II as a risk score that could be used as part of a triage system for resource allocation in patients with COVID-19. The aim of this work is to determine if the APACHE II is a risk score that performs well in patients with COVID-19 in times when resources are scarce and triage decisions need to be made and, if so, if it can be used as a tool to help guide those decisions.

## Materials and methods

This study is a single-center, longitudinal, retrospective observational cohort study. It was performed at Hospital Beatriz Ângelo, in Loures, Portugal. The study population includes all adult patients (18 years old or older) admitted to the ICU with COVID-19 between January 2020 and December 2021 with the APACHE II score evaluated upon admission. Patients were excluded from this study if they had a positive antigen test for influenza A or B virus or a positive polymerase chain reaction (PCR) test for influenza virus, they were admitted to the ICU for reasons other than COVID-19 or with asymptomatic COVID-19, they had a positive PCR test for severe acute respiratory syndrome coronavirus 2 (SARS-CoV-2) but were considered cured, and they are pregnant. COVID-19 patients are defined as patients with a positive PCR test for SARS-CoV-2 performed on nasopharyngeal swabs, tracheal secretions, or bronchoalveolar lavage.

During the study period, patients admitted to the ICU were treated according to local protocol and international guidelines. Stress ulcer prophylaxis with pantoprazole 40 mg intravenous (IV) daily was implemented. Venous thromboembolism prophylaxis was done with subcutaneous enoxaparin adjusted to the daily weight and renal function. Antibiotics were not routinely used unless patients had signs and symptoms suggestive of bacterial pneumonia or other bacterial infection. Empirical antibiotic treatment for bacterial pneumonia consisted either of ceftriaxone 2 g once daily IV or amoxicillin with clavulanic acid 2.2 g three times per day IV with azithromycin. When needed, patients receive ventilatory support. Ventilatory support started with a high-flow nasal cannula and noninvasive mechanical ventilation. Patients were sedated and intubated in case of respiratory fatigue, septic shock, or other indications. Mechanical ventilation was performed using acute respiratory distress syndrome protective ventilation guidelines. For sedation and analgesia propofol, fentanyl, midazolam, and ketamine were the main drugs used. Patients needing vasopressors were treated mainly with noradrenaline. Dexamethasone in a dose of 6 mg one time or two times per day was used in the majority of patients.

The primary endpoint was ICU mortality. The study population was divided into two periods: between November 2020 and February 2021 (from now on defined as Period 1), where the number of patients needing intensive care support outnumbered the available beds and resources, and between January and October 2020 and March and December 2021 (from now on defined as Period 2), where resources were adequate, as stated by the Portuguese health authorities [15,16]. Patients were followed until hospital discharge.

Data from patients were collected and analyzed retrospectively. The APACHE II score was calculated within the first 24 hours upon admission to the ICU. Sociodemographic and baseline characteristics were collected from the electronic medical records. Written informed consent was waived due to the anonymity of the data collected.

For the descriptive analysis of the baseline patients’ characteristics, continuous variables are reported as a mean and standard deviation (SD). Categorical variables are reported as frequencies. The normality of distribution was tested using the Kolmogorov-Smirnov test. Between-group comparisons were made using the Mann-Whitney rank-sum test for continuous variables and the chi-square or Fisher’s exact test for categorical variables. The AUROC was examined to identify the discriminative power of the APACHE II score to predict mortality in both periods. The predictive value is classified based on the AUROC as excellent discrimination (AUROC value: 0.99-0.9), very good (AUROC value: 0.89-0.8), good (AUROC value: 0.79-0.7), moderate (AUROC value: 0.69-0.6), or poor (AUROC value: <0.6) [22]. A logistic regression was used to determine the predictive value of the APACHE II score on the occurrence of death. Calibration was checked using the Lemeshow-Hosmer goodness-of-fit test. Checking the ROC curves and the associated tables, we determined the best cutoff value using the maximum Youden index. Based on that value, the patients were divided into two groups for both periods, those above and below the cutoff point. A Kaplan-Meier curve was then analyzed to compare the survival in both groups, and a log-rank test was used to check for differences between them. A two-sided P value of <0.05 was used to indicate statistical significance. Statistical analysis was performed using the Statistical Package for the Social Sciences (SPSS) version 26 for Windows (IBM SPSS Statistics, Armonk, NY, USA).

## Results

A total of 552 patients were admitted to the ICU of Hospital Beatriz Ângelo due to COVID-19 between January 2020 and December 2021. Of those patients, 124 were excluded from this analysis (two patients were excluded because they had a positive antigen test for influenza A or B virus, 26 were excluded because they were admitted to the ICU for reasons other than COVID-19, six were excluded because they had a positive PCR test for SARS-CoV-2 but were considered cured, and one was excluded because she was pregnant). The remaining 89 patients were excluded because the APACHE II score was not calculated upon admission or medical records did not have enough information to allow for its calculation.

Of the 428 patients included in the analysis, 229 were admitted to the ICU between November 2020 and February 2021, thus corresponding to Period 1, and the remaining 199 were admitted between January 2020 and October 2020, and March 2021 and December 2021, corresponding to Period 2. The mean age was 60.7 years old for both groups with 67.3% male patients. The mean APACHE II score value for the whole group was 12.13 points, 11.8 for Period 1 and 12.52 for Period 2. In total, 88 (20.6%) patients died in the ICU, 58 during Period 1 and 30 during Period 2. More detailed clinical and sociodemographic data and a descriptive analysis of patient characteristics can be found in Table 1.

**Table 1:** Baseline patients’ characteristics yr: year, SD: standard deviation, COPD: chronic obstructive pulmonary disease, ICU: intensive care unit, PaO2: partial pressure of oxygen, FiO2: inspired fraction of oxygen, APACHE II: Acute Physiology and Chronic Health Evaluation II, LOS: length of stay

	All (n = 428)	Period 1 (n = 229)	Period 2 ( n= 199)	P value
Clinical characteristics				
Age (yr), mean ± SD	60.7 ± 14.2	62.1 ± 12.3	59.1 ± 15.9	0.069
Age groups (yr), number (%)				
<50 years	91 (21.3)	37 (16.2)	54 (27.1)	
50-59 years	85 (19.9)	40 (20.5)	38 (19.1)	
60-69 years	124 (29)	73 (31.9)	51 (25.6)	
70-79 years	98 (22.9)	60 (26.2)	38 (19.1)	
>80 years	30 (7)	12 (5.2)	18 (9)	
Gender				
Male, number (%)	228 (67.3)	151 (65.9)	137 (68.8)	0.408
Female, number (%)	140 (32.7)	78 (34.1)	62 (31.2)	
Past medical history				
Hypertension, number (%)	246 (61.7)	143 (62.4)	121 (60.8)	0.728
COPD, number (%)	9 (2.1)	4 (1.7)	5 (2.5)	0.582
Cardiac disease, number (%)	35 (8.2)	25 (10.9)	10 (5)	0.027
Diabetes, number (%)	158 (36.9)	70 (30.6)	88 (44.2)	0.004
Malignancy, number (%)	27 (6.3)	14 (6.1)	13 (6.5)	0.859
Clinical signs and blood tests upon admission to the ICU				
Fever, number (%)	42 (9.8)	18 (7.9)	24 (12.1)	0.145
Mean arterial pressure (mmHg), mean ± SD	85.5 ± 19.9	83.8 ± 19.0	88.1 ± 20.8	0.029
Heart rate (beats/minute), mean ± SD	85.3 ± 21.6	84.34 ± 21.0	86.5 ± 22.3	0.396
Respiratory rate (breaths/minute), mean ± SD	27.3 ± 8.2	27.9 ± 8.4	24.7 ± 8.1	0.146
Acute renal failure, number (%)	120 (28)	63 (27.5)	57 (28.6)	0.795
Hematocrit (%), mean ± SD	37.8 ± 6.0	38.3 ± 5.7	37.3 ± 6.2	0.151
White blood cell count, mean ± SD	9.7 ± 4.8	9.7 ± 4.4	9.8 ± 5.3	0.498
PaO2/FiO2 (mmHg), mean ± SD	146.6 ± 92.4	136.3 ± 75.8	158.8 ± 107.3	0.021
APACHE II				
APACHE II score, mean ± SD	12.13 ± 5.94	11.8 ± 5.2	12.52 ± 6.7	0.434
Mortality risk % (APACHE II), mean ± SD	16.91 ± 13.8	16.3 ± 11.4	17.65 ± 16.2	0.607
Observed mortality, number (%)	88 (20.6)	58 (25.3)	30 (15.1)	0.009
ICU evolution				
LOS in the ICU, mean ± SD	9.0 ± 9.1	9.2 ± 10.0	8.8 ± 8.0	0.674
Noninvasive ventilation, number (%)	364 (85)	206 (90)	158 (79.4)	0.002
Mechanical ventilation, number (%)	219 (51.2)	117 (51.1)	102 (51.3)	0.973
Days under mechanical ventilation, mean ± SD	11.34 ± 9.9	12.5 ± 11.1	10.0 ± 8.2	0.182
Tracheostomy, number (%)	34 (7.9)	21 (9.2)	13 (6.5)	0.314
Renal replacement therapy, number (%)	31 (7.2)	19 (8.3)	12 (6.1)	0.374

**Figure 1:**
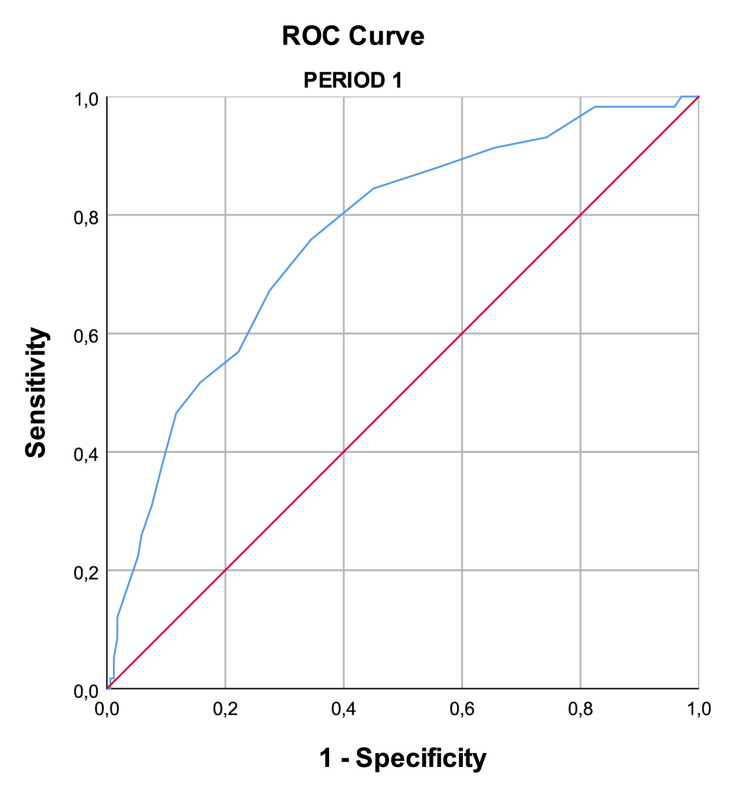
ROC curve (Period 1) The accuracy of the APACHE II score in patients admitted to the ICU in Period 1 (between November 2020 and February 2021), where the number of patients needing intensive care support outnumbered the available resources. The AUROC was 0.763, reflecting a good discriminative power. APACHE II: Acute Physiology and Chronic Health II, ICU: intensive care unit, AUROC: area under the receiver operating characteristic curve, ROC curve: receiver operating characteristic curve

**Figure 2:**
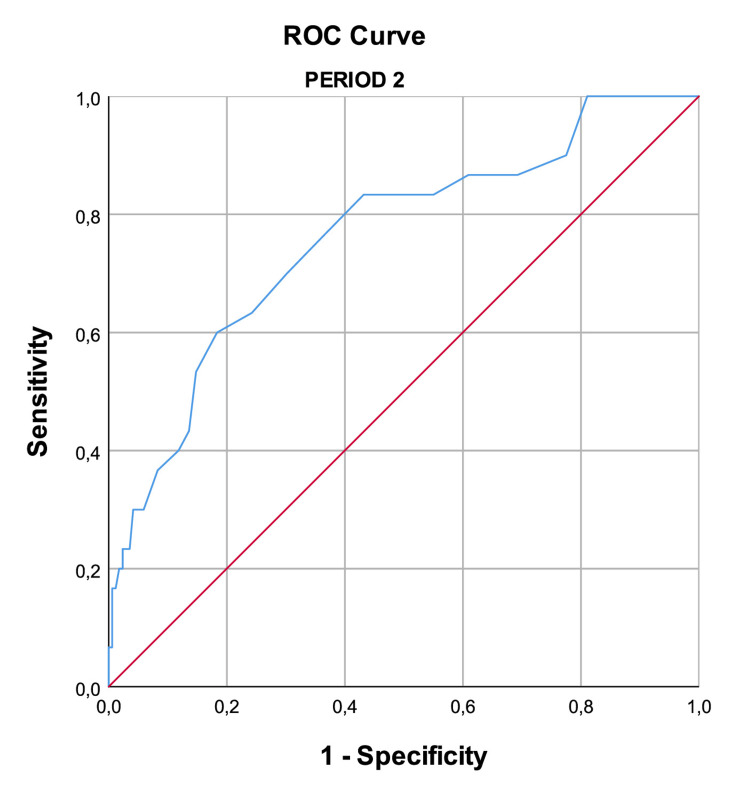
ROC curve (Period 2) The accuracy of the APACHE II score in patients admitted to the ICU in Period 2 (between January and October 2020, and March and December 2021), where ICU resources were adequate for the number of patients. The AUROC was 0.761, reflecting a good discriminative power. APACHE II: Acute Physiology and Chronic Health II, ICU: intensive care unit, AUROC: area under the receiver operating characteristic curve, ROC curve: receiver operating characteristic curve

The AUROC for Period 1 was 0.763 and for Period 2 was 0.761, reflecting a good discriminative power. Figure 1 and Figure 2, and Table 2 display those results.

**Table 2:** Discriminative power of the APACHE II AUROC values for the APACHE II score in Period 1 (between November 2020 and February 2021), where the number of patients needing intensive care support outnumbered the available resources, and Period 2 (between January and October 2020, and March and December 2021), where resources were adequate. AUROC: area under the receiver operating characteristic curve, APACHE II: Acute Physiology and Chronic Health Evaluation II, CI: confidence interval

	Area	Standard error	P value	95% CI	
				Lower bound	Upper bound
Period 1	0.763	0.036	<0.001	0.693	0.834
Period 2	0.761	0.050	<0.001	0.664	0.858

Calibration using the Hosmer-Lemeshow goodness-of-fit test is shown in Table 3. The APACHE II seems to be well calibrated for both periods (P > 0.05), although with a value of 0.925 for Period 1 and 0.175 for Period 2, suggesting that for Period 2, the agreement between the observed mortality and that predicted by the APACHE II score was lower than that for Period 1. Univariate logistic regression is also shown in Table 3. Both APACHE II scores for Period 1 and Period 2 were significant predictors of mortality in patients admitted to the ICU.

**Table 3:** Calibration and univariate logistic regression analysis Univariate regression analysis and calibration using the Hosmer-Lemeshow goodness-of-fit test of the APACHE II score. Both in Period 1 and Period 2, the APACHE II showed good calibration and was a significant predictor of mortality. APACHE II: Acute Physiology and Chronic Health Evaluation II, CI: confidence interval

	Hosmer-Lemeshow test		Univariate logistic regression			
			Exp(B)	95% CI for Exp(B)		P value
	Chi-square	P value		Lower	Upper	
Period 1	3.141	0.925	1.195	1.118	1.278	<0.001
Period 2	11.491	0.175	1.159	1.088	1.235	<0.001

**Figure 3:**
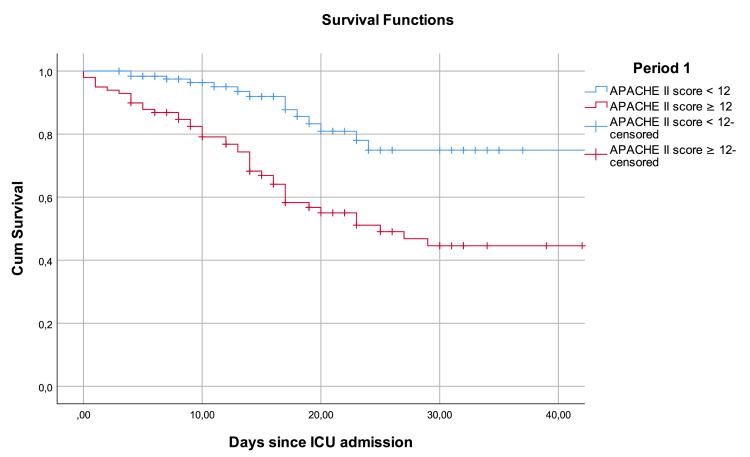
Kaplan-Meier analyses (Period 1) Kaplan-Meier survival curve of COVID-19 patients admitted to the ICU in Period 1 (between November 2020 and February 2021), where the number of patients needing intensive care support outnumbered the available resources, stratified by the APACHE II score. Log-rank test showed a chi-square of 18,196 with a P value of <0.001. COVID-19: coronavirus disease 2019, ICU: intensive care unit, APACHE II: Acute Physiology and Chronic Health Evaluation II

**Figure 4:**
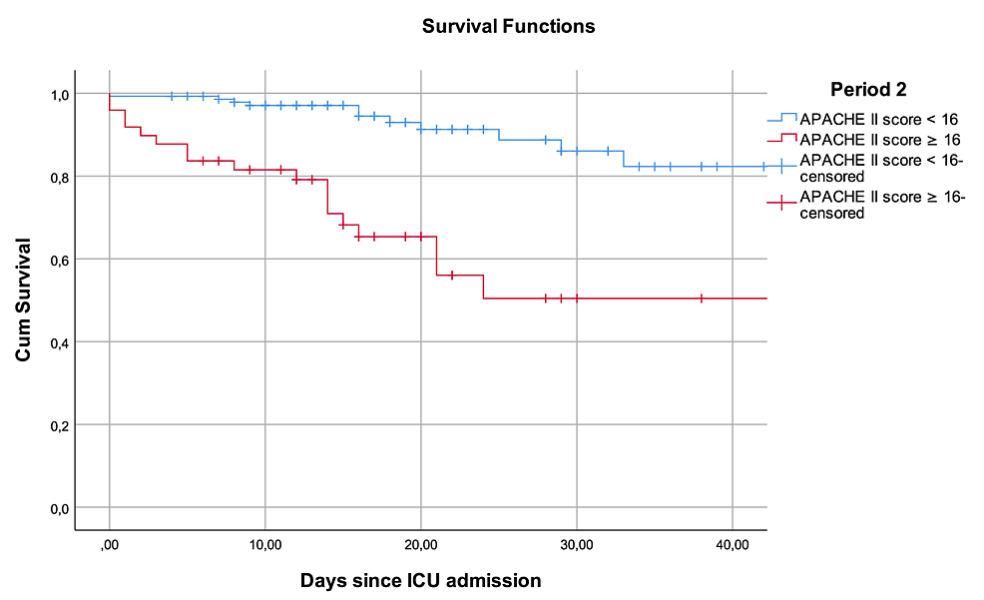
Kaplan-Meier analyses (Period 2) Kaplan-Meier survival curve of COVID-19 patients admitted to the ICU in Period 2 (between January and October 2020, and March and December 2021), where ICU resources were adequate for the number of patients, stratified by the APACHE II score. Log-rank test showed a chi-square of 22,114 with a P value of <0.001. COVID-19: coronavirus disease 2019, ICU: intensive care unit, APACHE II: Acute Physiology and Chronic Health Evaluation II

The Youden index was determined next. An APACHE II score value of 12 points or higher for Period 1 and 16 points or higher for period 2 corresponded to the best index of 0.414 and 0.417, respectively. For both periods, patients with APACHE II scores above those stated were associated with significantly higher mortality, compared to the ones with lower APACHE II scores, with a log-rank test showing a P value of <0.001. Figure 3 and Figure 4 represent the survival analysis using a Kaplan-Meier curve.

## Discussion

In this study, the APACHE II score showed a good discriminative power to predict mortality among patients admitted to the ICU with COVID-19 in a period with adequate resources to treat every patient and a period of resource scarcity where rational resource allocation was needed. The AUROC values of 0.763 and 0.761 are consistent with those observed in other studies validating the APACHE II score among patients with COVID-19 [8-13]. Regression analysis showed that the APACHE II score was associated with mortality in patients with COVID-19 in both periods as well. Survival analysis was consistent with the only other survival analysis for APACHE II in patients with COVID-19 available in the literature [8], showing a statistically significant difference in the survival of patients with low and high APACHE II score values during their stay at the hospital.

The calibration test demonstrated a good calibration for both periods, although lower for Period 2 than for Period 1. This finding might help explain why the observed mortality in Period 1 was higher than that in Period 2 (25.6% versus 15.1%), although the mean APACHE II score value and the AUROC of the risk score were similar. The fact that, in the period where resource allocation was not a concern, the calibration was lower can be explained by several factors. In that period, patients admitted to the ICU sometimes had therapeutic boundaries clearly defined (e.g., being candidates for noninvasive ventilation, but not being candidates for intubation), and patients were also admitted earlier in the course of the disease (sometimes admitted to the ICU if they had several predictors of COVID-19 severity [23], even without the need for intensive care at the moment).

What makes this study unique compared to the other studies mentioned above is that, so far, no other study tested the APACHE II score specifically in periods where patients with COVID-19 outnumbered the available ICU beds and resources. During the pandemic, several papers and guidelines discussed considerations regarding resource allocation, including the use of severity scores [17-21]. However, none of those considerations was based on actual data from COVID-19 patients, such as the one presented.

Prior to the current pandemic, prognostic risk scores were already part of triage protocols for ICU admission in times of resource scarcity, with proposed models based on ethical considerations, trained clinicians, and clinical decision support systems [24]. The inclusion of those risk scores in triage is thought to give some objectivity to the decision-making protocols [25]. Although some studies have looked at the values and use of the APACHE II during triage [5,7,26], a few studies have actually tried to validate its use in times when resources are scarce.

The APACHE II score is not only one of the most used risk scores worldwide to predict mortality in ICU patients but also one tool used for benchmarking, allowing ICU staff to improve the delivery of care [5,7]. Although newer APACHE scores have been developed [27,28], such scores are not available as a free tool for physicians, enhancing the importance of studies targeting the APACHE II.

This study has several possible limitations. First, it is a retrospective, single-center study as opposed to multicenter, prospective research that could provide more insight. During the two-year period that data were gathered, new guidelines were being issued on a regular basis, and the standard of care for COVID-19 patients was quickly evolving, meaning that not all patients included in this study were treated equally, possibly influencing the outcomes of those patients. Data for the study was collected from medical records that were elaborated by physicians during a period of both critical work conditions and mental fatigue. That translates into a certain degree of data deficiency and a considerable proportion of patients missing data to allow for the calculation of the APACHE II score, excluding them from this analysis.

Finally, it is important to discuss the implementation of the findings in clinical practice. Although this study validates the APACHE II in patients with COVID-19 in times when resources are scarce and a triage system is needed, due to the limitations mentioned above, some caution is needed when interpreting and applying the results. Multicenter studies with more patients would allow for more generalizable results. The APACHE II being a non-disease-specific prognostic scoring system also suggests that these findings from patients with COVID-19 could be similar to patients with other diseases, paving way for more and new research.

## Conclusions

The APACHE II score is an effective tool for predicting mortality in patients with COVID-19 admitted to the ICU in periods where resource allocation and triage of patients are needed. The APACHE II applied in COVID-19 patients in those periods showed a good discriminative power to predict mortality, and higher score values were associated with higher mortality. The results were similar when compared to periods where triage was not needed, and the results were also similar to those from past research.

The use of the APACHE II, combined with other ethical, clinical, and physical considerations, as well as the availability and training of healthcare personnel, may allow for the development of a better and improved triage system. Although several methodological limitations can be found, the findings from this study might help and stimulate the development of new research in this area to allow for a future clinical application.
